# Recursive computed ABC (cABC) analysis as a precise method for reducing machine learning based feature sets to their minimum informative size

**DOI:** 10.1038/s41598-023-32396-9

**Published:** 2023-04-04

**Authors:** Jörn Lötsch, Alfred Ultsch

**Affiliations:** 1 Institute of Clinical Pharmacology, Goethe - University, Theodor – Stern - Kai 7, 60590 Frankfurt am Main, Germany; 2 Fraunhofer Institute for Translational Medicine and Pharmacology ITMP, Theodor – Stern - Kai 7, 60596 Frankfurt am Main, Germany; 3 DataBionics Research Group, University of Marburg, Hans – Meerwein – Straße 22, 35032 Marburg, Germany

**Keywords:** Machine learning, Software, Statistical methods

## Abstract

Selecting the k best features is a common task in machine learning. Typically, a few features have high importance, but many have low importance (right-skewed distribution). This report proposes a numerically precise method to address this skewed feature importance distribution in order to reduce a feature set to the informative minimum of items. Computed ABC analysis (cABC) is an item categorization method that aims to identify the most important items by partitioning a set of non-negative numerical items into subsets "A", "B", and "C" such that subset "A" contains the "few important" items based on specific properties of ABC curves defined by their relationship to Lorenz curves. In its recursive form, the cABC analysis can be applied again to subset "A". A generic image dataset and three biomedical datasets (lipidomics and two genomics datasets) with a large number of variables were used to perform the experiments. The experimental results show that the recursive cABC analysis limits the dimensions of the data projection to a minimum where the relevant information is still preserved and directs the feature selection in machine learning to the most important class-relevant information, including filtering feature sets for nonsense variables. Feature sets were reduced to 10% or less of the original variables and still provided accurate classification in data not used for feature selection. cABC analysis, in its recursive variant, provides a computationally precise means of reducing information to a minimum. The minimum is the result of a computation of the number of k most relevant items, rather than a decision to select the k best items from a list. In addition, there are precise criteria for stopping the reduction process. The reduction to the most important features can improve the human understanding of the properties of the data set. The cABC method is implemented in the Python package "cABCanalysis" available at https://pypi.org/project/cABCanalysis/.

## Introduction

Selecting a few relevant items from a ranked list is a common task in data analysis and the key task in feature selection for machine learning^1^. Feature selection can be seen, first, as the calculation of the importance of each feature (variable) of a multivariate dataset and, second, as the selection of the "important few". The feature importance calculation should be performed by a problem specific method, such as correlations (PCS), non-normality (ICA), methods that read the feature importance from trained classifiers, and many others.

Typically, the distribution of feature importance is highly skewed, i.e., a few variables have high importance, but many have low importance. Reducing a data set to the most relevant variables can be a goal of the analysis in several ways: to reduce computational cost, to reduce the cost of data acquisition, to increase statistical power, to eliminate non-informative variables that reduce the performance of the algorithms, or to reduce the number of variables in knowledge discovery to a number that enhances human understanding of the relevant items, to the point of highlighting the key process responsible for the class structure in a data set.

In the context of knowledge discovery by feature selection, the underlying assumption is that if an algorithm can be trained with information (variables) to assign a case to the correct class, then the variables needed for this success contain relevant information about the class structure being addressed. However, too much information in too many variables can prevent domain experts from grasping the main mechanistic processes underlying a class structure in a data set, and they will tend to lose the interpretation in the vast knowledge of many details of the research domain that is abundantly available to specialized experts.

This report therefore addresses a reductionist approach to feature selection and knowledge discovery, i.e., reducing a problem as close as possible to a key question or a main causal reason. A numerically precise method is proposed for reducing a feature set to its informative minimum of items. It is based on item or inventory categorization by the so-called computed ABC (cABC) analysis method^2^ and aims at reducing a feature set to its minimum informative size, which provides sufficient information for successful training of a classifier.

## Methods

### Ambiguous meaning of the term "ABC analysis”

A search of the PubMed database using the web interface at https://pubmed.ncbi.nlm.nih.gov/ on May 31, 2022 for "ABC analysis" (with quotation marks) returned 103 hits. However, the term "ABC analysis" is used ambiguously in the biomedical literature. "ABC analysis" is used for: (i) "Ap-proximate Bayesian Computation" (e.g.,^3,4^), (ii) "Antecedent-Behavior-Consequence", which is a method for analyzing behavior and comes from the field of psychology^5^, and (iii) an inventory categorization method originating in economics, where ABC occasionally stands for "Always Better Control", used in inventory control and management (e.g.,^6–9^).

This report refers only to the idea underlying the latter meaning of ABC analysis, i.e., the item or inventory categorization method that aims to identify the most relevant items by dividing a set of non-negative numerical items into subsets "A", "B", and "C"^10^. However, the term "Always Better Control" for "ABC", which was never used by Juran, should not be associated with the present method. Instead, "ABC" refers to an analogy with U.S. school grades, where the grade "A" is the best, which is consistent with the present meaning, where the items assigned to the subset "A" can be considered the best, i.e., "A grade" items. ABC analysis deals with skewed distributions and exploits their properties rather than trying to normalize them. Specifically, ABC analysis as inventory or item categorization is concerned with the unequal importance of individual inventory items, where a few important items account for most of the total inventory, while many other items are of such low value that they have little impact on the results.

**Figure 1:**
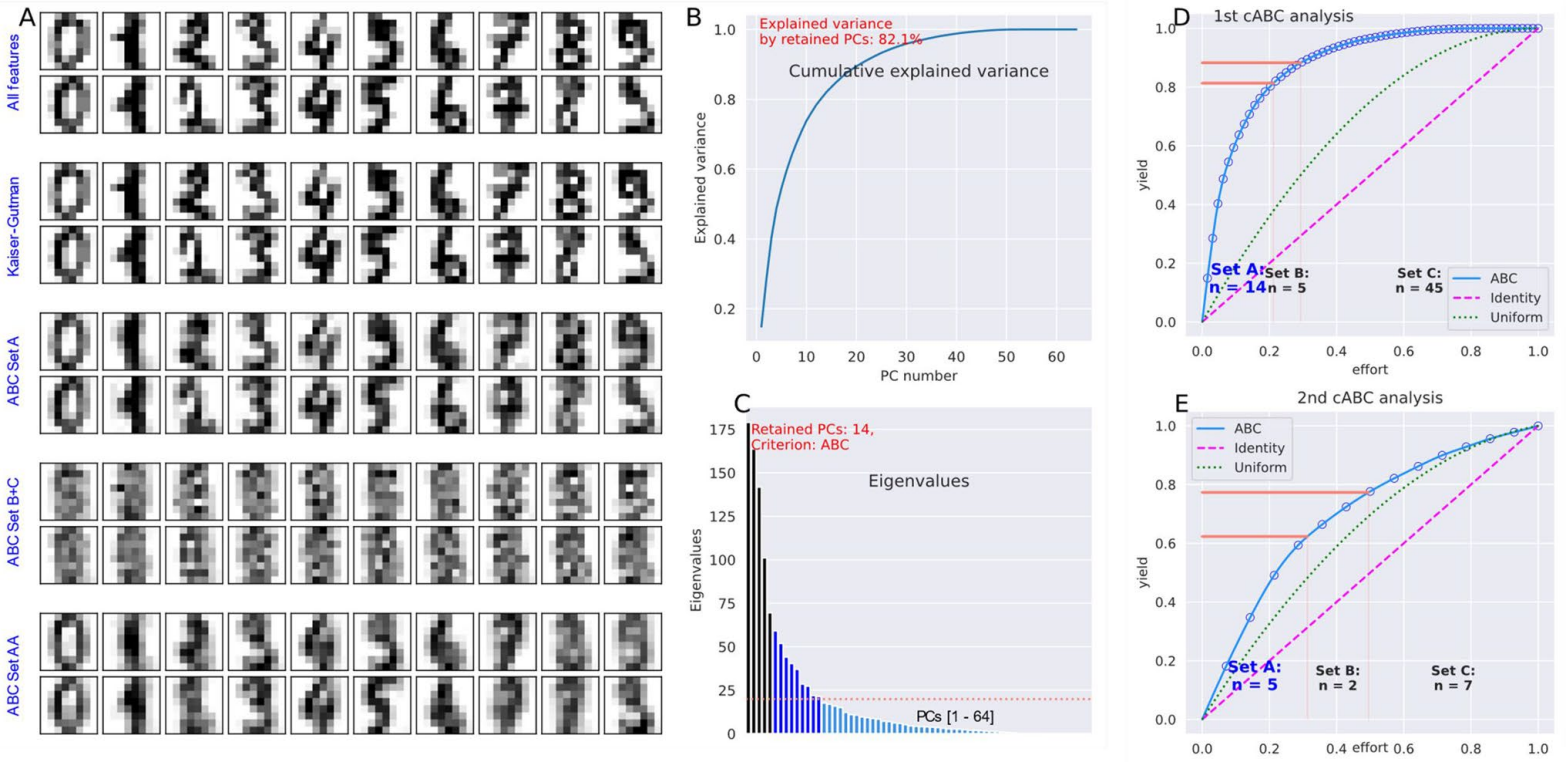
Comparison of information reduction performance between cABC analysis and Kaiser-Guttman criterion (eigenvalues > 1) for correlation-based PCA weighting of features in pixel gray values of 8 × 8 images of handwritten digits. The image gray value information was taken from the dataset collection provided with the "scikit-learn" package (https://scikit-learn.org/stable/^40^). (**A**) 16 example images of handwritten digits using (i) the original 8 × 8 pixels, (ii) reproduction based on the d = 47 PCs with eigenvalues > 1, (iii) reproduction based on the d = 14 PCs selected as “important few” by the first cABC analysis, (iv) reproduction based on the PCs that had not been selected by the cABC analysis, and (v) reproduction based on the d = 5 PCs selected by the recursive 2nd cABC analysis. (**B**) line plot of cumulative explained variance with increasing number of principal components (PCs). (**C**) Bar graph of the eigenvalues of the individual PCs. Bar colors indicate selection in different repeated selection steps using cABC analysis, from light blue = "not selected" to dark blue and black for features selected in the first and second cABC analysis. (**D**) and (**E**) Results of the cABC analysis of the mean variable importance. The ABC plots (blue lines) show the cumulative distribution function of the importance variables together with the identity distribution, x_i_ = constant (magenta line), and the uniform distribution, i.e., as a stopping criterion for the repetitions of the cABC analysis. The red lines show the boundaries between the ABC subsets "A", "B" and "C". The figure has been created using Python version 3.8.13 for Linux (https://www.python.org) and Seaborn Python data visualization library (https://seaborn.pydata.org^22^) and our Python package "cABCanalysis" (https://pypi.org/project/cABCanalysis/).

ABC analysis can be represented graphically by plotting the (positive) importance values sorted from largest to smallest. The cumulative sum of the largest values is plotted against the fraction of values that are summed (see, for example, Fig. 1D or Fig. 3B). If the order of the values is reversed to least to greatest, the ABC curve is identical to the Lorentz curve. Many ABC curves appear to pass through a point where 80% of the cumulative sum of the largest values has been achieved with 20% of the items. This is often incorrectly referred to as the "Pareto 80/20" principle. However, the cutoffs are not clearly specified in the literature on ABC analysis. One of the proposals assigns the 20% of the items that make up 70% of the total value to the subset "A"^11^, others propose the 10% of the items that make up 2/3 of the total value^12^, or the classical "Pareto" cut-off (80/20) is used. Common to all these approaches is the arbitrary definition of the cut-off value for the boundaries between subsets "A" and "B" and between subsets "B" and "C".

### Computed ABC (cABC) analysis

Recently, the term "computed ABC analysis" has been introduced for an item categorization technique that replaces the arbitrary definition of the set limits by a well-founded algorithmic calculation from the data^2^. The details can be summarized as follows. The graphical presentation of the cumulative item contributions, sorted from the most contributing to the least contribution item, on a xy-coordinate system is an “ABC curve” where non-negative values x_i_ are sorted in decreasing order: (for all i) *x*_*i*_ ≥ *x*_*i*+ 1_. The fraction of the first *i* items to *n*, *E*_*i*_ = *i/n*, represents costs or “efforts”, *E*_*i*_. The fraction of the cumulative sum of the *x*_*i*_, relative to the total sum, is called the “yield”, *Y*_*i*_, of *x*_*1*_*, …x*_*i*_ obtained as ${Y}_{i}= \frac{\sum_{k=1}^{i}x_{k}}{{\sum }_{k=1}^{n}x_{k}}$. The items *x*_*1*_, …, *x*_*k*_ denote a set of n positive values (*x*_*i*_ > 0). The points (X_i_, Y_i_) are interpolated using splines^13^. ABC curves are particularly meaningful when the distribution of the values *x*_*i*_ is uneven, i.e., few *x*_*i*_ have very large values while many *x*_*i*_ have only small values. The ABC curve, originally called the Lorenz curve^13^, is a special form of a graphical representation of cumulative distributions^14,15^.

The "Pareto" point [x, y] = [0, 1] is the ideal point where no effort (x = 0) delivers all the yield (y = 1). The point on the ABC curve, which lies nearest to this point (“Juran” point) is then a real existing combination of effort and yield that comes as close as possible to the ideal “Pareto” point. In cABC analysis, the “Juran” point (J_x_, J_y_) is calculated as the point with the smallest distance from points on the ABC curve to the “Pareto” point. From these considerations it follows that J_x_ is well suited to mark the boundary between subsets "A" and "B". Furthermore, the so-called "break-even increment" is calculated via the first derivative of the ABC curve, where the slope $\frac{dy}{dx}ABC$= 1. At this point, an increase in effort invested is equivalent to an increase in profit earned. The boundary between subsets "B" and "C" (BC boundary) is calculated in the same way on the ABC curve as for the AB point, where the ABC curve starts at the AB boundary. Thus, computed ABC (cABC) analysis is a classification technique for asymmetric, positive-valued distributions.

### Recursive computed ABC analysis

#### Motivation

In some contexts, the goal of feature selection is to reduce the size of the most important items (subset "A") to very few. For example, in knowledge discovery, it may be desirable to reduce the number of relevant variables to a human-understandable set to facilitate interpretability. A suggestion for the size of such a set was made about 70 years ago by Miller, who proposed a size of 7 ± 2 as the optimal number for human understanding^16^. Algorithmically, this can be achieved by performing the cABC analysis recursively. This means that the cABC analysis is applied to the items that were assigned to subset "A" in a previous cABC analysis, creating set A within set A. The recursively computed ABC analysis creates smaller and smaller subsets of items consisting of more and more important items.

#### Termination criteria for recursive cABC analysis

Three criteria are defined for stopping the repetition of the cABC analysis on the previous "A" subset of features:

1. A first criterion is the classification performance of the algorithms trained with the selected features, which should be satisfactory. It should not drop significantly from the performance achieved with all features and should at least be better than chance.
2. A second criterion is a theoretical limit for selecting the best items from a set. The recursive ABC analysis ends when the distribution of the items is uniform. Any compact subset (consecutive sequence of items) of a uniform distribution is also uniformly distributed. Therefore, the uniform distribution is a fixed point of ABC analysis. Note that in the uniform distribution, all values that *x*_*i*_ can take have the same probability of having a value in the interval from *a* = *min(X)* to *b* = *max(X)*. The uniform distribution is different from the identity distribution, where all items have the same numerical value. However, the latter is sometimes confused with the former, even in some data science tutorials. The ABC curve *ABC*_*uniform*_*(p)* of a uniform distribution within the limits [0, b] is given as *ABC*_*uniform*_*(p)* = *− p*^*2*^ + *2p*. For the uniform distribution, the AB limit can be calculated analytically as 41% of the items (details not shown). Therefore, reducing the number of features with uniform distribution of their importance measure results in the top 41% to be selected, which can be done manually without the need to calculate a cABC analysis.
3. A third criterion is contextual. If the feature set is considered sufficiently reduced, even though criteria 1 or 2 for termination do not yet apply, the selection can be terminated. Recursive cABC analysis can also be used to reduce any set of items to the size of the above-mentioned "Miller optimum" of 7 ± 2.

### Implementation in Python

The implementation in the Python programming language^17^ was done as the Python package "cABCanalysis", which is available at https://pypi.org/project/cABCanalysis/. The Python package imports parts of the Python packages "pandas" (https://pandas.pydata.org^18,19^), the numerical Python package "numpy" (https://numpy.org^20^), basic algorithms for scientific computing in Python "SciPy" (https://scipy.org^21^), and the graphical frameworks of the Python data visualization package "Seaborn" (https://seaborn.pydata.org^22^) and the graphics package "matplotlib" (https://matplotlib.org^23^).

#### Validation of the Python package

The present Python implementation "cABCanalysis" of the computed ABC analysis (https://pypi.org/project/cABCanalysis/) was compared with the R implementation in our R library "ABCanalysis" (https://cran.r-project.org/package=ABCanalysis), using the same examples as in the publication of the raw method and its R implementation^2^. Selected common data distributions were analyzed (Table 1). Differences between the Python and R implementations in the number of items assigned to the ABC subset "A" or to the fixed point of the cABC analysis for the uniform distribution of 41% of the items were analyzed using Wilcoxon–Mann–Whitney U tests^24,25^. This showed that the sizes of subset "A" did not differ significantly for the current selection of χ2, lognormal, exponential, Pareto, and uniformly distributed data; however, for the Gaussian distribution, the Python implementation always assigned 41% of the data to subset "A", while the R implementation produced set sizes slightly below this value, and the difference was statistically significant (Table 2). However, the differences were mostly less than 1%. However, the percentage of items in subset "A" depended on the total number of items. For a small number of n = 10 or n = 100 items, the sizes of subset "A" between the Python and R implementations were perfectly or largely matched (Table 2), but the percentage of total items converges to a fixed value per sample distribution only for larger set sizes, e.g., 1000 or 10,000. A likely explanation for the slight differences in the results is the use of spline interpolation routines imported from different packages, i.e. "scipy.interpolate.CubicSpline" from the Python package "SciPy" or the R base package "stats". It is known that results obtained with different software packages often do not agree slightly^26^.

**Table 1:** The cABC analysis was applied to data sets with n = 1000 instances generated with selected popular distributions.

Distribution	Size of subset A (mean ± SD [%]		Difference Python—R	*p*-value of difference Python—R	*p*-value of the difference to 41%	
	Python	R			Python	R
${{\varvec{\chi}}}_{1}^{2}$	28.4 ± 0.7	28.4 ± 0.5	0.03 ± 0.27	0.85	0.005	0.006
Log-normal N (0, 3)	7.2 ± 2.1	7.1 ± 2.4	0.09 ± 0.36	0.97	0.006	0.002
Exponential (β = 1)	33.1 ± 0.5	33.1 ± 0.5	0.04 ± 0.38	0.7	0.004	0.006
Pareto (α = 1.18)	14.5 ± 4	14.3 ± 3.7	0.2 ± 0.31	0.85	0.006	0.006
Uniform [0, 100]	41 ± 0.5	40.9 ± 0.3	0.08 ± 0.32	0.73	1	0.413
Gaussian N (5, 1)	46.6 ± 0	46.5 ± 0.2	0.14 ± 0.15	0.012	0.002	0.006

Comparison of the sizes of the ABC subset "A" obtained with the Python or R implementations of the computed ABC (cABC) analysis, available as packages "cABCanalysis" (https://pypi.org/project/cABCanalysis/) and "ABCanalysis" (https://cran.r-project.org/package=ABCanalysis^2^), respectively. In addition, the difference of the obtained values is compared to the value of 41% assigned in the subset "A" from the whole sample, as a fixed point of the ABC curve for the uniform distribution. The *p-*values of the differences between the two implementations are the results of Wilcoxon–Mann–Whitney U tests^24,25^ against each other or against a fixed value of 41%, which is the fixed point for ABC analysis.

**Table 2:** The cABC analysis was applied to data sets with n = [10, 100, 1000, 10,000] instances generated with selected distributions.

Distribution	n = 10		n = 100		n = 1000		n = 10,000	
	Python	R	Python	R	Python	R	Python	R
${{\varvec{\chi}}}_{1}^{2}$	34 ± 5.2	33 ± 4.8	29.3 ± 1.5	28.6 ± 1.4	28.4 ± 0.7	28.4 ± 0.5	28.4 ± 0	28.1 ± 0.2
Log-normal N (0, 3)	25 ± 9.7	23 ± 8.2	10.3 ± 4.7	9.3 ± 4.7	7.2 ± 2.1	7.1 ± 2.4	6.5 ± 2.54	6.6 ± 2.1
Exponential (β = 1)	34 ± 5.2	33 ± 6.7	34.3 ± 1.8	33.2 ± 1.6	33.1 ± 0.5	33.1 ± 0.5	33.4 ± 0	33.2 ± 0.1
Pareto (α = 1.18)	28 ± 6.3	28 ± 6.3	21.6 ± 4.9	20.6 ± 4.9	14.5 ± 3.9	14.3 ± 3.7	14.8 ± 2.32	14.7 ± 2.3
Uniform [0, 100]	43 ± 6.7	42 ± 7.9	41.3 ± 1.2	40.5 ± 1.3	41 ± 0.5	40.9 ± 0.3	41.5 ± 0	41.1 ± 0.1
Gaussian N (5, 1)	50 ± 4.7	49 ± 3.2	47 ± 0.5	46.5 ± 0.7	46.6 ± 0	46.5 ± 0.2	46.7 ± 0	46.5 ± 0.1

The set sizes of the ABC subset "A" obtained using either the Python or R implementations of the computed ABC analysis (cABC), available as packages "cABCanalysis" (https://pypi.org/project/cABCanalysis/) and "ABCanalysis" (https://cran.r-project.org/package=ABCanalysis^2^), are given as a function of the number of instances. The mean percentages of items assigned to subset "A" and the standard deviations of these percentages are given.

### Experimentation

Programming was done using Python version 3.8.13, freely available at https://www.python.org (accessed June 2, 2022). Experiments were performed in the Anaconda data science environment (Anaconda Inc., Austin, TX, USA), freely available at https://www.anaconda.com), on an AMD Ryzen Threadripper 3970X (Advanced Micro Devices, Inc., Santa Clara, CA, USA) computer running Ubuntu Linux 22.04.1 LTS (Canonical, London, UK).

For the present experiments, random forests^27,28^ were used as the basis for feature weighting. It reportedly outperformed other machine learning based methods^29^ and offers some advantages over alternatives. Random forest classifiers inherently allow feature importance estimation through permutation weighting^28^ of out-of-bag (OOB) cases, as the classification accuracy decreases when the particular feature is omitted from the class assignment. Random forests are considered to be powerful classifiers, on tabular numerical data even compared to deep learning neural networks^30,31^, and have been shown to outperform logistic regression^32^ and does not require sophisticated variable transformations or scaling. Possible supervised alternatives have more drawbacks, e.g. the k-nearest neighbors classifier (kNN)^33^ requires a valid distance measure, which can be difficult to define^34^, support vector machines (SVM)^35^) have a critical and difficult to set hyperparameter, regularization^36^, which should be set by experts and therefore makes SVM inappropriate for the current focus of feature selection, and deep learning layered artificial neural networks (ANN), while being universal classifiers^37^, have two parameters that are difficult to set, namely the number of layers and the number of neurons in each layer.

Random forests and feature selection based on cABC analysis of their importance for classifier training were performed after setting aside 20% of the subjects, proportional to the classes of patients and controls, as a validation sample that was not touched until classifier training was complete. The data of the remaining 80% of the cases were used to train the classifier using the RandomForestClassifier method from the sklearn.ensemble module of the scikit-learn Python package. After hyperparameter tuning of the classifier^38^ (for code details, see Table 3), the feature selection methods were applied in a 5 × 20 nested cross-validation scenario provided by the method "RepeatedStratifiedKFold" from the module "sklearn.model_selection" of "scikit-learn", with the parameters "n_splits" = 5 and "n_repeats" = 20. During each cross-validation run, feature importance was computed using the generic permutation importance provided by the "permutation_importance" method of the "sklearn.inspection" package, with the number of permutations set to n_repeats = 50. The cABC analysis was then performed on the average of the feature importance measures calculated in each run. Note that the classifiers were always retuned to the actual data sets.

**Table 3:**
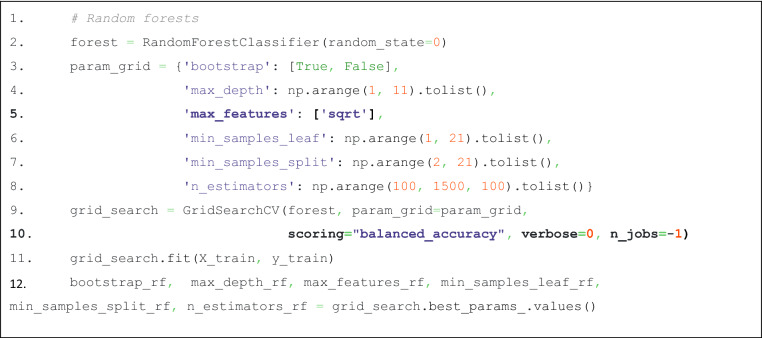
Python code used for the tuning of the hyperparameters of the random forest classifiers.

To evaluate the usefulness of the selected features for classification, as well as to estimate the consequences of reducing the number of variables for training/validation, random forest classifiers were trained on the training data subset, using the full and reduced feature sets obtained from the recursive cABC analyses, using a 5 × 20 nested cross-validation scenario. During these nested cross-validation runs, classifier performance was quantified by calculating the balanced class assignment accuracy in randomly drawn 80% of the 20% validation data set separated at the beginning of the data analysis^39^. The bounds of the non-parametric 95% confidence interval of the balanced accuracy were set as the 2.5th and 97.5th percentiles of the values obtained in the cross-validation runs. This interval should not include the value of 0.5 (50%) balanced accuracy, because then the class assignment cannot be considered better than by guessing.

#### Images data set of handwritten digits

Feature selection with computed ABC analysis was evaluated on a dataset of handwritten digits from the dataset collection provided with the "scikit-learn" package (https://scikit-learn.org/stable/^40^). It contains about 180 8 × 8 pixel images of handwritten digits [0, …, 9] each, with a total size of 1797 × 64 data points. Features were selected based on the importance of the d = 64 variables in a principal component analysis (PCA)^41,42^ projection onto uncorrelated principal planes. The number of relevant principal components (PCs) to retain was selected by applying cABC analysis to the eigenvalues. The remaining PCs were overwritten with zeros and the projected data were back-transformed into the original data space.

#### Lipidomics data set for Parkinson's disease

The Parkinson's disease lipidomics dataset includes plasma concentrations of d = 25 lipid markers studied in samples from n = 100 Parkinson's disease patients and n = 100 healthy controls and was previously described in^43^. The aim of the original study was to investigate the regulation of lipid signaling and lipid marker patterns in Parkinson's disease. Lipid marker concentrations were rescaled to the range [0, 100] from a previous analysis where marker patterns were sought^43^. The use of computational ABC analysis in combination with variable importance computation in training machine learning algorithms has already been described for this dataset using the R implementation^44^.

#### Genomics data set for leukemia

A data set designed to demonstrate the feasibility of cancer classification based solely on gene expression monitoring is available in the R package "golubEsets" (https://bioconductor.org/packages/golubEsets^45^). The data set^46^ has an original size of 72 × 7130 and consists of expression data of 7129 genes analyzed with Affymetrix Hgu6800 chips from bone marrow samples of two patient classes, i.e. 47 patients with acute lymphoblastic leukemia (ALL) and 25 patients with acute myeloid leukemia (AML; class information). For the present experiments, the first 150 gene expression data were used, sorted in decreasing order of variance as suggested in http://rstudio-pubs-static.s3.amazonaws.com/3773_0afaead59a02436889abc68753e6c20a.html. Feature selection, classifier training and performance testing for the leukemia data set were performed as described above for the Parkinson's disease data set.

#### Genomics data set for high opioid dosing requirements

The "high opioid dosage requirements" dataset^47^ contains next-generation sequencing (NGS)-derived exonic sequences of the opioid receptor genes *OPRM1*, *OPRK1*, *OPRD1*, and *SIGMAR1*, located on chromosomes 6, 8, 1, and 9, respectively, which encode µ-, κ-, and δ-opioid receptors, as well as the intracellular sigma-nonopioid receptor, commonly considered a member of the opioid receptor family. The dataset contains d = 152 variants in opioid receptor genes obtained from n = 64 patients treated with opioid analgesics for persistent pain. In addition, d = 19 additional gene loci from the vicinity of the opioid receptor gene sequences remained in the data set. For the present demonstration, subjects were grouped according to their need for high or usual doses of opioids at a threshold of 500 mg oral morphine equivalents (OME) per day. This resulted in n = 38 patients being assigned to the "usual" opioid dose group and n = 27 patients requiring "very high" doses. Feature selection, classifier training and performance testing were performed as described above.

#### Sensorics and genomics data for pain

Pain-related data were available from an assessment of sensitivity to experimental noxious stimuli and the genomic background of pain collected in a quantitative sensory testing study in n = 125 healthy young volunteers^48^. The dataset includes subject sex, pain thresholds to heat, cold, blunt pressure, puncture pressure, and electrical stimuli with and without prior sensitization by topical application of capsaicin or menthol cream, and genetic information on 29 common variants in eight human genes reported to modulate pain, including single nucleotide variants and haplotypes. For the present assessments, the task was defined to assign the sex of the subjects (69 males, 56 females) from the acquired sensory and genetic information, reversing the generally accepted fact that pain perception is sex-specific^49^.

### Ethical approval and consent to participate

Not applicable. Data have been taken from publicly available sources.

## Results

### Recursive cABC analysis limits the dimensions of the PCA projection to a minimum while preserving relevant information

#### Images data set of handwritten digits

The computed ABC analysis provided a useful method for selecting the relevant principal components to retain in the PCA for further analysis. Using cABC analysis of eigenvalues to determine the number of relevant PCA components resulted in k = 14 components to retain (Fig. 1B), which is a substantial reduction in information from the k = 47 components retained using the commonly used Kaiser-Guttman criterion of eigenvalues > 1^50,51^. Nevertheless, the information was sufficient to recognize the digits in the images created with the back-transformed retained PCs. On the other hand, if only the information from the PCs that were not to be retained was back-transformed, the images no longer reproduced the original information of the digits. The information reduction could be continued in the recursive cABC analysis until only k = 5 PCs could be used to reconstruct still recognizable images of handwritten digits. Feature selection was stopped after the second cABC analysis because the eigenvalues of the remaining 5 PCs were uniformly distributed (Kolmogorov–Smirnov test against uniform distribution: *p* = 0.341), i.e. the second stopping criterion for the recursive cABC analysis was fulfilled. The numerical results of the classification experiments are shown in Table 4. They confirm that up to the second cABC analysis, the features contained enough information to assign an image to the correct numerical digit with accuracies close to 1. Only 8% of the variables (PCs) resulting from the recursive cABC analysis were sufficient for this task, while the standard Kaiser-Gutman criterion reduced the number of variables to only 73%.

**Table 4:** Feature selection using recursive cABC analysis on the eigenvalues of principal components in the PCA projection of pixel gray values in 8 × 8 images of handwritten digits and the performance of random forest classifiers, quantified as balanced accuracy (and 95% nonparametric confidence interval, CI).

cABC times	KS-test *p*-value for item list	Number of features (% of all)	Median balanced accuracy (95% CI) (validation data)	Features
0	6.476·10^–31^	64 (100%)	0.97(0.96–0.98)	All PCs
0	3.072·10^–18^	Kaiser-Gutman: 47 (73%)	0.97(0.97–0.99)	PC 1—47
1	0.00047	A: 14 (22%)	0.97(0.96–0.98)	PC 1—14
2	0.341	AA: 5 (8%)	0.91(0.89–0.93)	PC 1—5
2	–	AA: 5	0.50(0.42–0.57)	As above but target assignment permuted
1	–	BC: 40 (62%)	0.57(0.53–0.6)	PC 15—64

For comparison, the commonly used Kaiser-Guttman criterion of eigenvalues > 1^50,51^ was applied to select the relevant PCs. The data was taken from the Python package "scikit-learn" (https://scikit-learn.org/stable/^40^) and contains about 180 8 × 8 pixel images of handwritten digits [0, …, 9] each, with a total size of 1797 × 64 data points. The classification task was to assign the handwritten digits to the correct number. In addition, the *p*-values of a Kolmogorov–Smirnov test^60^ of the distribution of the values subjected to cABC analysis against the uniform distribution are reported. Classification accuracy refers to the 20% validation sample not used for feature selection and classifier training.

### Recursive cABC analysis guides machine learning feature selection to the most important class-relevant information

#### Lipidomics data set for Parkinson's disease

**Figure 2:**
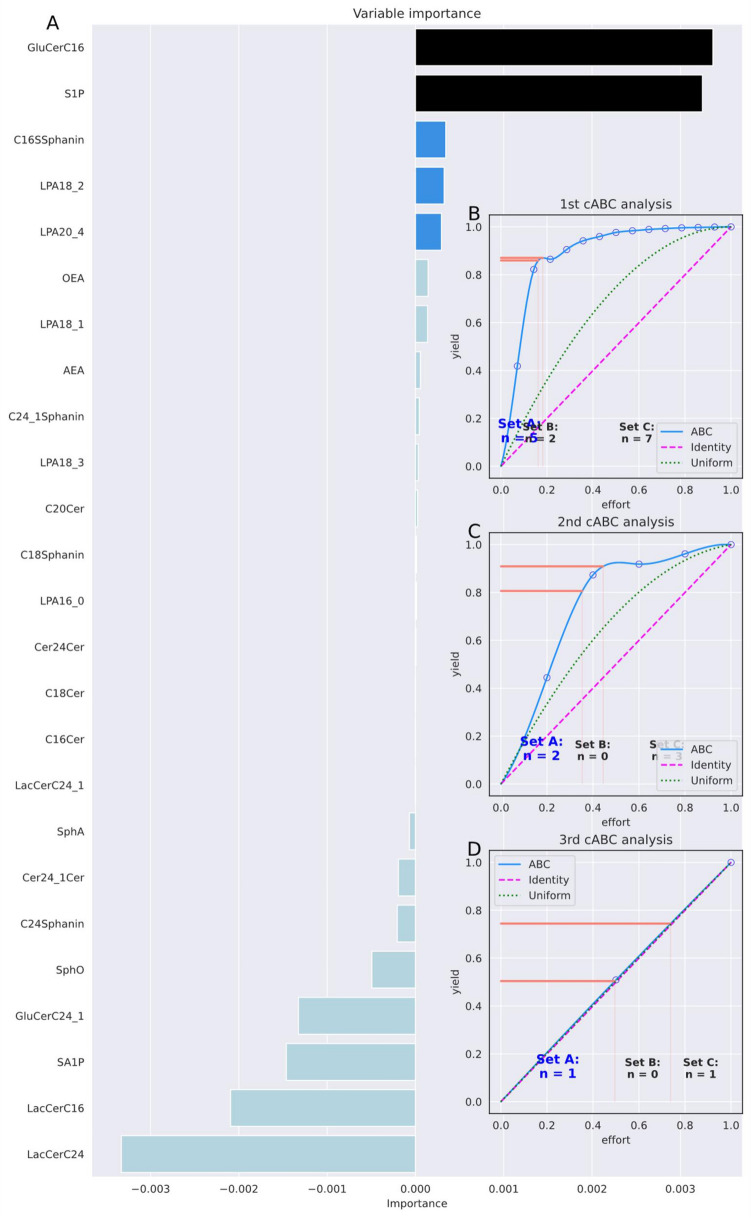
Feature selection using recursive cABC analysis for Parkinson's disease lipidomics data. The dataset consisted of d = 25 lipid markers measured in the plasma of n = 100 Parkinson patients and healthy controls, respectively^43,44^. (**A**) Variable importance according to a 5 × 20 nested cross-validation feature selection using random forests and the generic permutation importance provided in the "permutation_importance" method of the "sklearn.inspection" package. Bar colors indicate the selection of informative variables in different repeated selection steps using cABC analysis, from light blue = "not selected" to dark blue and black for features selected in deeper until the last repetition of cABC analysis. (**B**) and (**C**) Results of the cABC analysis of the mean variable importance. The ABC plots (blue lines) show the cumulative distribution function of the importance variables together with the identity distribution, x_i_ = constant (magenta line), and the uniform distribution, i.e., as a stopping criterion for the repetitions of the cABC analysis. The red lines show the boundaries between the ABC subsets "A", "B" and "C". The figure was created using Python version 3.8.13 for Linux (https://www.python.org) with the seaborn statistical data visualization package (https://seaborn.pydata.org^22^) and our Python package "cABCanalysis" (https://pypi.org/project/cABCanalysis/).

Of the original d = 25 lipid markers, random forests required only one, sphingosine-1-phosphate (S1P), to identify a blood sample as coming from a patient with Parkinson's disease (PD) or a healthy control subject (Fig. 2). The recursive ABC analysis further reduced the selected feature set to d = 5 and finally to only d = 1 lipid markers (Table 5). One glucosylceramide (GluCerC16) still provided enough information for perfect class assignment. The results are consistent with previous findings obtained with other data analysis methods^43,52^ and are not discussed further in the present method report. However, in another study, GluCerC16 was also the best discriminator between multiple sclerosis patients and controls^53^, suggesting that it is a general marker for neurodegenerative disease rather than specific for PD.

**Table 5:** Feature selection using recursive cABC analysis in the Parkinson's disease lipidomics data set and performance of random forest classifiers, quantified as balanced ac accuracy (and 95% nonparametric confidence interval, CI).

cABC times	KS-test *p*-value for item list	Number of features (% of all)	Median balanced accuracy (95% CI) (validation data)	Features
0	5.769·10^−14^	25 (100%)	1 (1–1)	AEA, OEA, LPA16_0, LPA18_1, LPA18_2, LPA18_3, LPA20_4, C16Cer, C18Cer, C20Cer, Cer24Cer, Cer24_1Cer, GluCerC16, GluCerC24_1, LacCerC16, LacCerC24, LacCerC24_1, SphA, SA1P, SphO, S1P, C16SSphanin, C18Sphanin, C24Sphanin, C24_1Sphanin
1	0.037	A: 5 (20%)	1 (1–1)	LPA18_2, LPA20_4, GluCerC16, S1P, C16SSphanin
2	0.002	AA: 2 (8%)	1 (1–1)	GluCerC16, S1P
3	–	AAA: 1 (4%)	0.97 (0.94–1)	GluCerC16
3	–		0.52 (0.32–0.7)	As above but target assignment permuted

The data set consisted of d = 25 lipid markers measured in the plasma of n = 100 Parkinson patients and healthy controls, respectively^43,44^. During feature selection, the cABC analysis was applied recursively to the items assigned to ABC subset "A" in the previous run, starting with the full feature set. Recursive subsets are named "AA", "AAA", etc. In addition, the *p*-values of a Kolmogorov–Smirnov test^60^ of the distribution of the values subjected to cABC analysis against the uniform distribution are reported. Classification accuracy refers to the 20% validation sample not used for feature selection and classifier training.

#### Genomics data set for leukemia

**Figure 3:**
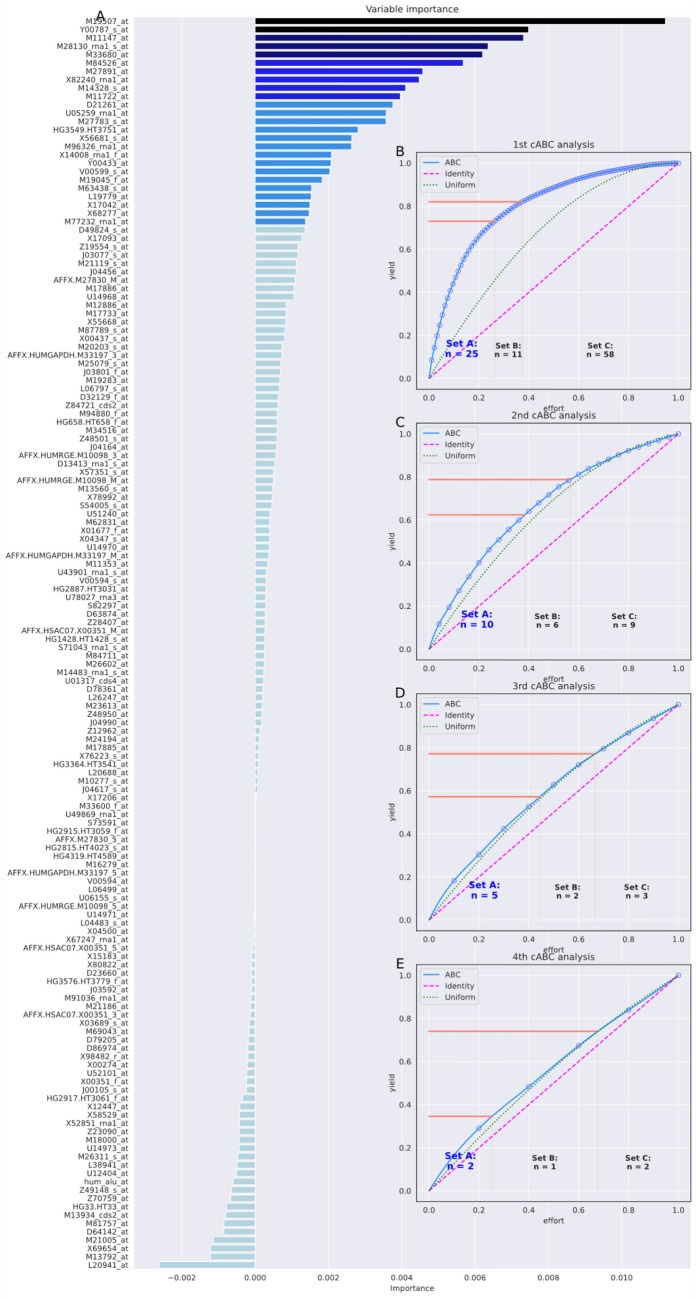
Feature selection using recursive cABC analysis in the genomics dataset for leukemia. (https://bioconductor.org/packages/golubEsets^45^). The data set consisted of expression data of d = 150 genes, queried from n = 47 patients with acute lymphoblastic leukemia (ALL) and n = 25 patients with acute myeloid leukemia (AML)^45,46^. (**A**) Variable importance according to a 5 × 20 nested cross-validation feature selection using random forests and the generic permutation importance provided in the "permutation_importance" method of the "sklearn.inspection" package. Bar colors indicate the selection of informative variables in different repeated selection steps using cABC analysis, from light blue = "not selected" to dark blue and black for features selected in deeper until the last repetition of cABC analysis. (**B**)–(**D**) Results of the cABC analysis of the mean variable importance. The ABC plots (blue lines) show the cumulative distribution function of the importance variables together with the identity distribution, x_i_ = constant (magenta line), and the uniform distribution, i.e., as a stopping criterion for the repetitions of the cABC analysis. The red lines show the boundaries between the ABC subsets "A", "B" and "C". The figure was created using Python version 3.8.13 for Linux (https://www.python.org) with the seaborn statistical data visualization package (https://seaborn.pydata.org^22^) and our Python package "cABCanalysis" (https://pypi.org/project/cABCanalysis/).

Feature selection in the leukemia data set (Fig. 3) was able to reduce the number of variables to d = 25 that could be used to train a random forest classifier that could assign a patient to either ALL or AML diagnosis with a perfect balanced accuracy of 1 (Table 6). The recursive ABC analysis further reduced the selected feature set to d = 10, 5, and finally to only d = 2 genes. Even with this small set, the classification performance was perfect in the 20% validation sample drawn before feature selection. The analysis was stopped at this stage using the third stopping criterion, i.e., the feature set was considered small enough and the cost of information reduction was evident in the drop in balanced accuracy to 86%.

**Table 6:** Feature selection using recursive cABC analysis in the leukemia genomics dataset (https://bioconductor.org/packages/golubEsets^45^), quantified as balanced accuracy (and 95% nonparametric confidence interval, CI).

cABC times	KS-test *p*-value for item list	Number of features (% of all)	Median balanced accuracy (95% CI) (validation data)	Features
0	5.71·10^–19^	150 (100%)	1 (1–1)	All d = 150 genetic markers
1	0.00032	A: 25 (16.7%)	1 (1–1)	M63438_s_at, M11147_at, M19507_at, M27891_at, M96326_rna1_at, M27783_s_at, L19779_at, Y00787_s_at, X56681_s_at, Y00433_at, V00599_s_at, X82240_rna1_at, D21261_at, M14328_s_at, X68277_at, M28130_rna1_s_at, X14008_rna1_f_at, M19045_f_at, M84526_at, HG3549.HT3751_at, M77232_rna1_at, M33680_at, M11722_at, X17042_at, U05259_rna1_at
1			0.5 (0.21–0.83)	As above but target assignment permuted
2	0.0336	AA: 10 (6.7%)	1 (1–1)	M11147_at, M19507_at, M27891_at, Y00787_s_at, X82240_rna1_at, M14328_s_at, M28130_rna1_s_at, M84526_at, M33680_at, M11722_at']
3	0.00557	AAA: 5 (3.3%)	0.88 (0.75–1)	M11147_at, M19507_at, Y00787_s_at, M28130_rna1_s_at, M33680_at
4	0.00287	AAAA: 2 (1.3%)	0.86 (0.75–1)	M19507_at, Y00787_s_at

The data set consisted of expression data of d = 150 genes sequenced from n = 47 patients with acute lymphoblastic leukemia (ALL) and n = 25 patients with acute myeloid leukemia (AML)^45,46^. Classification accuracy refers to the 20% validation sample not used for feature selection and classifier training. The genetic marker annotations correspond to the annotation data of the Affymetrix Hu6800 array (chip hu6800); for details see e.g. https://bioconductor.org/packages/hu6800.db/). The cABC analysis was applied recursively ("recursive cABC analysis") to the items assigned to ABC subset "A" in the previous run, starting with the full feature set. Recursive subsets are named "A", "AA", etc. In addition, the *p*-values of a Kolmogorov–Smirnov test^60^ of the distribution of the values subjected to cABC analysis against the uniform distribution are reported.

#### Genomics data set for high opioid dosing requirements

**Figure 4:**
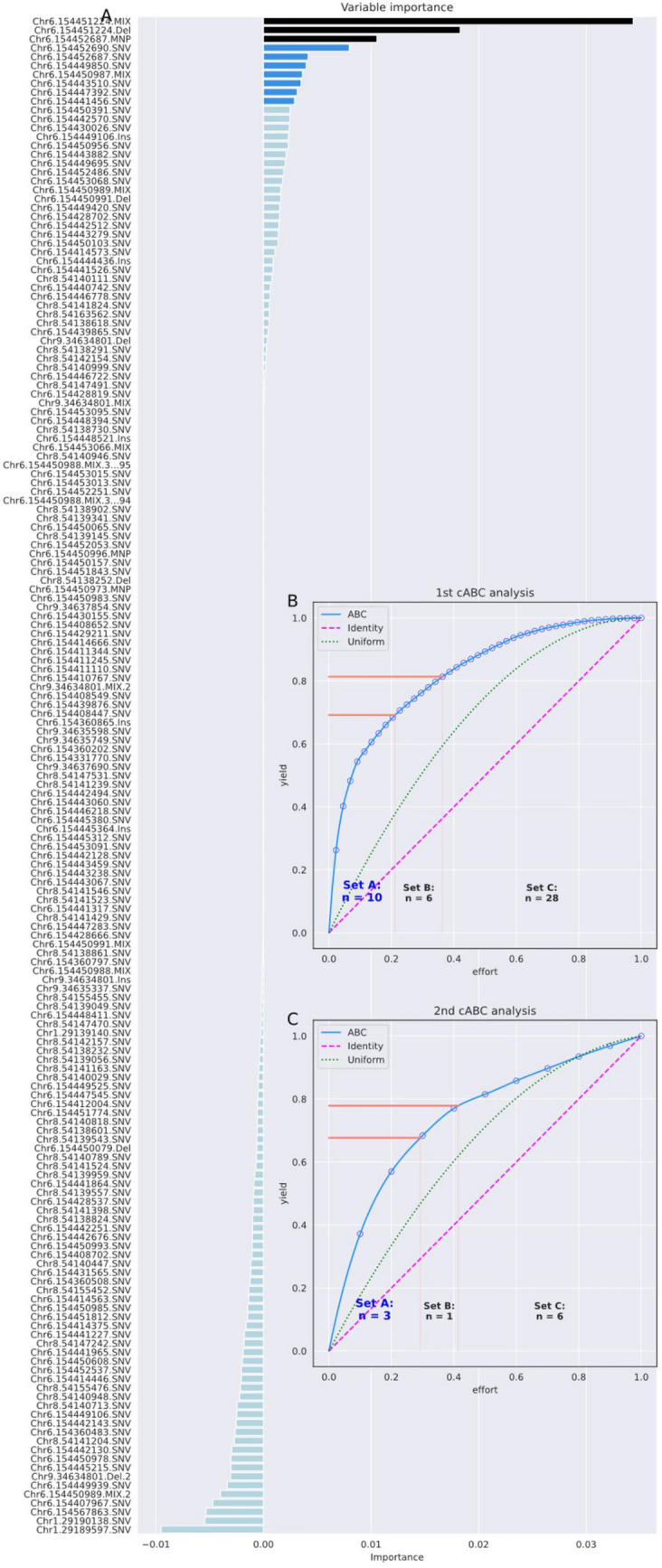
Feature selection using recursive cABC analysis in the high opioid dose requirement dataset consisting of d = 171 variants in the opioid receptor genes *OPRM1*, *OPRK1*, *OPRD1*, and *SIGMAR1*, located on chromosomes 6, 8, 1, and 9, respectively, and adjacent regions, acquired in n = 64 patients treated with opioid analgesics for persistent pain, n = 38 at "usual" opioid doses, and n = 27 at very high doses^47^. (**A**) Variable importance according to a 5 × 20 nested cross-validation feature selection using random forests and the generic permutation importance provided in the "permutation_importance" method of the "sklearn.inspection" package. Bar colors indicate the selection of informative variables in different repeated selection steps using cABC analysis, from light blue = "not selected" to dark blue and black for features selected in deeper until the last repetition of cABC analysis. (**B**) and (**C**) Results of the cABC analysis of the mean variable importance. The ABC plots (blue lines) show the cumulative distribution function of the importance variables together with the identity distribution, x_i_ = constant (magenta line), and the uniform distribution, i.e., as a stopping criterion for the repetitions of the cABC analysis. The red lines show the boundaries between the ABC subsets "A", "B" and "C". The figure was created using Python version 3.8.13 for Linux (https://www.python.org) with the seaborn statistical data visualization package (https://seaborn.pydata.org^22^) and our Python package "cABCanalysis" (https://pypi.org/project/cABCanalysis/).

The recursive cABC analysis ended with d = 3 loci in the *OPRM1* gene (Fig. 4), when the second stopping criterion was applied because the importance measure was uniformly distributed (Table 7). One of the three variants in the final feature set, Chr6.154451224.MIX, was found only six times in the entire cohort and only in subjects with high opioid requirements of > 500 mg/d OME. The variant has not yet been characterized for its functional significance for the µ-opioid receptor.

**Table 7:** Feature selection using recursive cABC analysis in the genomics dataset for high opioid dosage requirements, quantified as balanced accuracy (and 95% nonparametric confidence interval, CI).

cABC times	KS-test *p*-value for item list	Number of features (% of all)	Median balanced accuracy (95% CI) (validation data)	Features
0	6.35·10^–58^	171 (100%)	0.66 (0.52–0.83)	All d = 171 genetic markers
1	0.0011	A: 10 (5.8%)	0.74 (0.53–0.9)	Chr6.154441456.SNV, Chr6.154443510.SNV, Chr6.154447392.SNV, Chr6.154449850.SNV, Chr6.154450987.MIX, Chr6.154451224.Del, Chr6.154451224.MIX, Chr6.154452687.SNV, Chr6.154452687.MNP, Chr6.154452690.SNV
2	0.481	AA: 3 (1.8%)	0.79 (0.7–0.88)	Chr6.154451224.Del, Chr6.154451224.MIX, Chr6.154452687.MNP
3	–		0.48 (0.3–0.71)	As above but target assignment permuted

The data set consisted of d = 171 variants in the opioid receptor genes *OPRM1*, *OPRK1*, *OPRD1*, and *SIGMAR1*, located on chromosomes 6, 8, 1, and 9, and adjacent regions, acquired from n = 64 patients treated with opioid analgesics for persistent pain, n = 38 on "usual" opioid doses, and n = 27 on very high doses. Classification accuracy refers to the 20% validation sample not used for feature selection and classifier training. The cABC analysis was applied recursively ("recursive cABC analysis") to the items assigned to ABC subset "A" in the previous run, starting with the full feature set. Recursive subsets are named "A", "AA", etc. In addition, the *p*-values of a Kolmogorov–Smirnov test^60^ of the distribution of the values subjected to cABC analysis against the uniform distribution are reported.

### Recursive cABC analysis provides a data-driven means of filtering feature sets for nonsense variables

#### Sensorics and genomics data for pain

**Figure 5:**
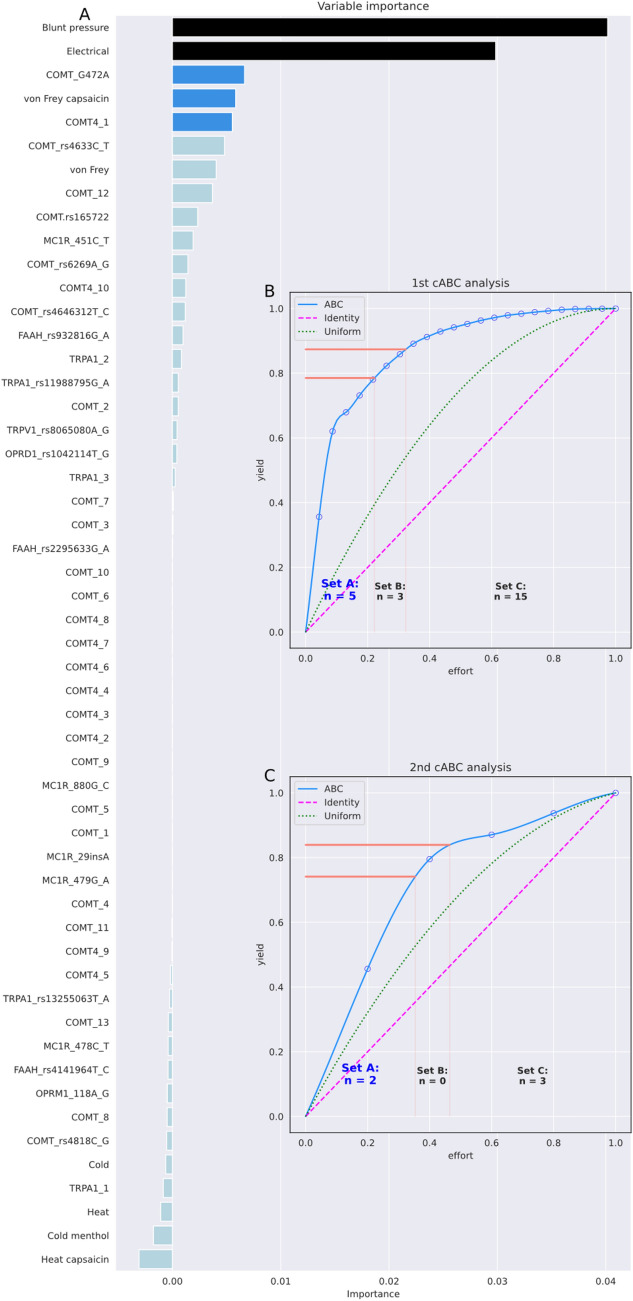
Feature selection using recursive cABC analysis in sensory and genomic data for pain. The dataset includes subject gender, pain thresholds to heat, cold, blunt pressure, punctate pressure (von Frey hairs), and electrical stimuli with and without prior sensitization by local application of capsaicin or menthol cream, and genetic information on 29 common variants in eight human genes reported to modulate pain, including single nucleotide variants and haplotypes, acquired from n = 125 healthy young volunteers^48^. (**A**) Variable importance according to a 5 × 20 nested cross-validation feature selection using random forests and the generic permutation importance provided in the "permutation_importance" method of the "sklearn.inspection" package. Bar colors indicate the selection of informative variables in different repeated selection steps using cABC analysis, from light blue = "not selected" to dark blue and black for features selected in deeper until the last repetition of cABC analysis. (**B**) and (**C**) Results of the cABC analysis of the mean variable importance. The ABC plots (blue lines) show the cumulative distribution function of the importance variables together with the identity distribution, x_i_ = constant (magenta line), and the uniform distribution, i.e., as a stopping criterion for the repetitions of the cABC analysis. The red lines show the boundaries between the ABC subsets "A", "B" and "C". The figure was created using Python version 3.8.13 for Linux (https://www.python.org) with the seaborn statistical data visualization package (https://seaborn.pydata.org^22^) and our Python package "cABCanalysis" (https://pypi.org/project/cABCanalysis/).

An assessment of the importance of sensory and genetic information in the pain-related data set suggests that, at best, sex can be inferred from pain threshold to mechanical blunt pressure and to some extent from electrical stimuli (Table 8). However, feature selection initially identified d = 5 variables as highly relevant to sex segregation, including genetic variables in the catechol-O-methyltransferase gene, namely COMT G472A and a haplotype composed of four of the single nucleotide polymorphisms tested in this study. This seems unjustified, as it would imply that the variants are systematically more common in one of the sexes, rather than just a random distribution in the selected cohort that was not matched for genotypes. The second cABC analysis solved this problem by reporting only d = 2 sensory variants (Fig. 5).

**Table 8:** Feature selection using recursive cABC analysis in sensory and genomic data for pain, quantified as balanced accuracy (and 95% nonparametric confidence interval, CI).

cABC times	KS-test *p*-value for item list	Number of features (% of all)	Median balanced accuracy (95% CI) (validation data)	Features
0	1.61·10^–28^	53 (100%)	0.64 (0.53–0.75)	All d = 52 sensorics variables and genetic markers
1	0.044	6 (11.3%)	0.69 (0.59–0.8)	“von Frey hairs plus capsaicin”, “blunt pressure”, “electrical”, “COMT_G472A”, “COMT4_1”
2	0.132	2 (3.8%)	0.7 (0.57–0.8)	“blunt pressure”, “electrical”,
2	–	2	0.55 (0.3–0.72)	As above but target assignment permuted

The dataset includes subject sex, pain thresholds for heat, cold, blunt pressure, punctate pressure (von Frey hairs), and electrical stimuli with and without prior sensitization by topical application of capsaicin or menthol cream, and genetic information on 29 common variants in eight human genes reported to modulate pain, including single nucleotide variants and haplotypes, obtained from n = 125 healthy young volunteers^48^. Classification accuracy refers to the 20% validation sample not used for feature selection and classifier training. The cABC analysis was applied recursively ("recursive cABC analysis") to the items assigned to ABC subset "A" in the previous run, starting with the full feature set. Recursive subsets are named "A", "AA", etc. In addition, the *p*-values of a Kolmogorov–Smirnov test^60^ of the distribution of the values subjected to cABC analysis against the uniform distribution are reported.

## Discussion

When large data values are observed with much lower probability than small values, a meaningful subset of values may contain most of the information. Such distributions are often observed in biology, medicine, and other fields. Lognormal or other types of power transformations are common methods to make a distribution Gaussian, i.e., normally distributed. However, there are uses and interpretations of the untransformed skewed distributions. The cABC analysis can directly address skewed distributions in terms of information theory^5^. The recursive application of item categorization, implemented as cABC analysis, reduced large feature sets to a bare minimum that still allowed random forest classifiers to be trained to perform classification of new data with high accuracy. Alternatively, it provided computed bounds to reduce the amount of information to a fraction that still adequately captures the whole truth, as in the example of PCA reconstruction of images of handwritten digits. Thus, the method provides a tool for reducing both computational and data acquisition costs. Moreover, by drastically reducing the number of features, it can be a suitable basis for knowledge discovery in biomedical data. Importantly, the minimum size is the result of computing the number of k relevant items, rather than a decision to select the k best items from a list.

One problem with selecting items from a ranked list is defining a well-reasoned cutoff. Cutoffs are often set heuristically. For example, in PCA, the number of components that explain 95% of the total variance (e.g.^54^) or only those with eigenvalues > 1^50,51^. However, arbitrary values are also commonly used, for example in the Python implementation of the so-called "SelectKBest" feature selection method for machine learning, available in the "sklearn.feature_selection" module of the "scikit-learn" Python package (https://scikit-learn.org/stable/^40^). This will return features according to the k highest scores, but the value of k must be set to a certain number. There are techniques that can be used to avoid arbitrary choices, such as grid search-based approaches to "select k best" feature selection. However, these approaches depend on the particular algorithm, while a more general approach to item categorization and selection of the most important variables would be desirable. The exact computation of well-founded set bounds in the form of computational ABC analysis is a general approach applicable to all types of positive numerical data, with utility in skewed distributions such as feature importance in machine learning.

Computational ABC analysis, and especially nested cABC analysis, are precise algorithmic implementations of the principle of parsimony, also known as Occam's razor. This is the problem-solving principle that in the case of competing theories or explanations, the simpler one, e.g., a model with fewer parameters, should be preferred. In science, parsimony is an important heuristic for arriving at appropriate theories. Knowledge discovery by applying feature selection techniques to complex data sets assumes that if a classifier can be trained to assign a patient to the correct class better than by guessing, then the variables needed by the classifier to accomplish this task contain relevant information about the class structure being addressed. In this way, the most informative variables can be identified. In this use of feature selection, creating a powerful classifier is not the end goal, but feature selection takes precedence over classifier performance.

In this context, the feature selection algorithm is tasked with extracting information from the data set to present to the domain expert for further scientific interpretation. Depending on the context, the expert's understanding can be enhanced by both broadening and narrowing the feature set. An example of expanding the thematic focus is recognizing various different psychological traits, such as depression or anxiety, as relevant to the development of persistent pain, based on the occurrence of several items of the corresponding questionnaires in a set of selected features^55^. On the other hand, narrowing the focus by reducing the number of responses from 75 in the full psychological questionnaire battery, which is difficult to interpret item by item, to only seven better captures the important picture that emerges from reducing complexity to only relevant information about specific psychological features in the context of pain^56^. Deciding what level of complexity reduction provides the best knowledge discovery and understanding of the underlying biomedical problem is primarily a decision for the expert in the field, and data analysis can provide suggestions to support this decision-making process.

### Strengths and limitations

ABC curves, and therefore cABC analysis, are invariant to scaling. This means that multiplying the data by any non-zero factor will not change the analysis. However, ABC methods have the limitation that they are not translation invariant, i.e., adding a value to all items changes the ratio of the largest to the smallest data and thus the results of the ABC methods with respect to the set limits. In the ABC methods discussed here, this is addressed by normalizing the data points so that the minimum of the data is equal to zero. Like any ABC analysis, the cABC analysis is limited to non-negative efforts (items, data values). The basis of the analysis, the ABC curve, is a representation of the relative concentration in the data. It is most useful for right-skewed distributions. For such distributions, there are only a few items (data points) that account for most of the cumulative sum of all values.

**Figure 6:**
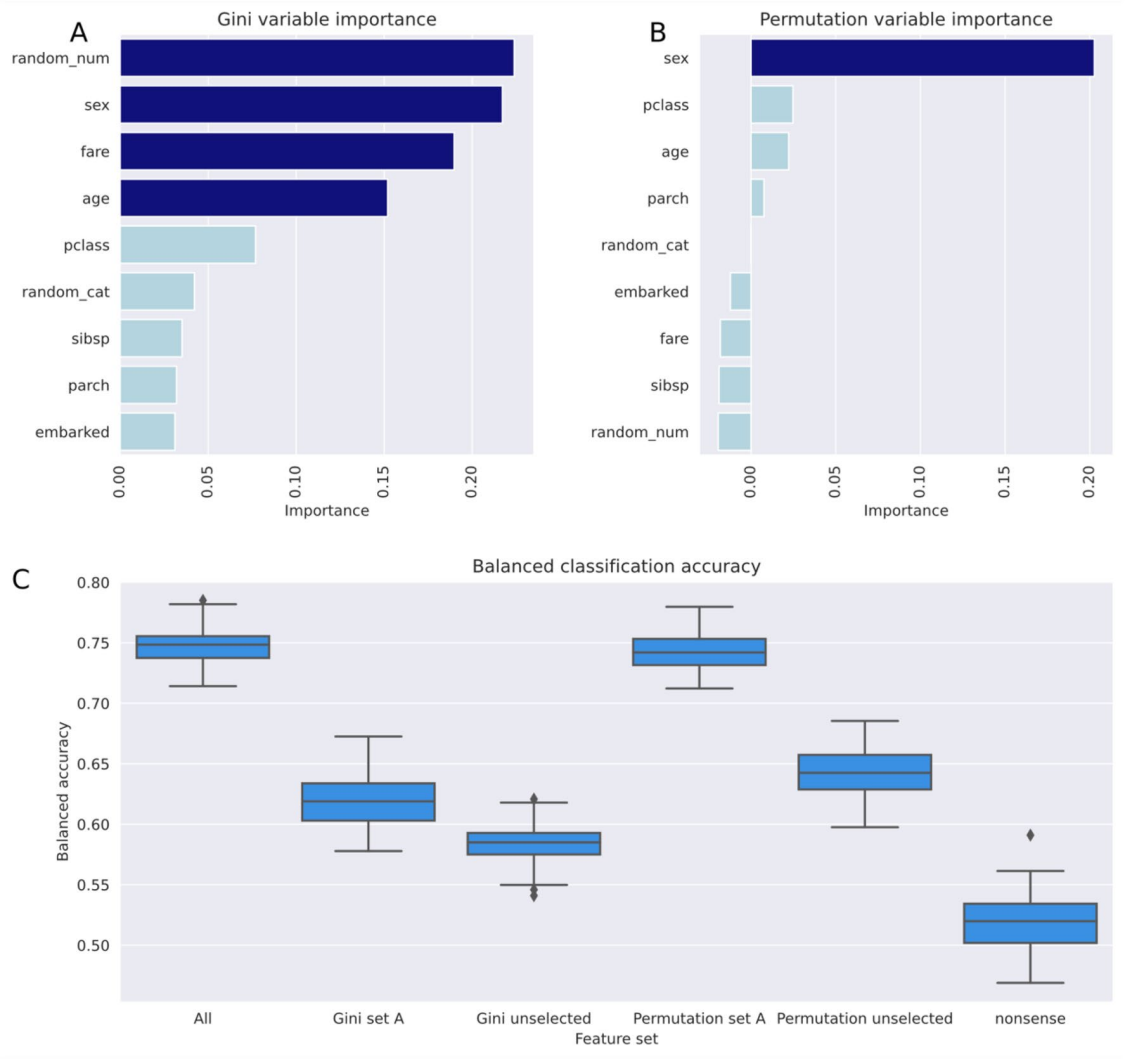
Example of a feature set when the selection methods works erroneously, modified from the documentation of the "scikit-learn" package (https://scikit-learn.org/stable/^40^) using code provided at https://scikit-learn.org/stable/auto_examples/inspection/plot_permutation_importance.html. The data set consists of variables of interest to the sinking of the Titanic, download via fetch_openml ("titanic", version = 1, as_frame = True, return_X_y = True, parser = "pandas"), with the addition of two random variables (“random_cat” and “random_num”). (**A**) Partially inflated variable importance according to the Gini impurity-based calculation. Bar colors indicate the selection of important variables using cABC analysis, from light blue = "not selected" to dark blue for selected features. (**B**) Variable importance according to the recommended permutation importance-based calculation. (**C**) Random forests classifier performance, expressed as balanced accuracy, using the selected features from the respective ABC subsets "A", the unselected features from the ABC subsets "B" and "C", and the added random nonsense variables. The example was run in 100 cross-validations. The figure was created using Python version 3.8.13 for Linux (https://www.python.org) with the seaborn statistical data visualization package (https://seaborn.pydata.org^22^).

The second stopping criterion, the uniform distribution of the item sets subjected to cABC analysis, is a tested criterion that depends on the statistical method chosen. For the present experiments, the Kolmogorov–Smirnov test was chosen. It should be noted, however, that the results of other tests may not be consistent when the *p*-value is close to 0.05. However, continuing the analysis with uniformly distributed items only selects the next 41% of the best items, which does not seem to cause major problems or generate "spurious" features. It just weakens the cutoff for recursion. The results of the digits dataset experiment show that reducing the information in the feature selection can come at a cost, i.e., the image quality in the present example was lower than when all PCs were reprojected. The selected features were able to represent the most important information in the data, i.e., the digits were still recognizable in the reprojected images. The loss of quality is perhaps most obvious in the first "5" (Fig. 1), which was already hard to distinguish from a "9" in the original image, and became even harder to distinguish in the reprojected images.cABC analysis addresses positive data and is particularly useful for right-skewed distributions, typical of feature sets where only a few of many variables are highly relevant to the structure of a data set. It provides a formal solution to the classic "Pareto 80/20" principle by computing the exact bounds between subsets. Inherently, it cannot provide a correction for feature importance measures assigned by a feature selection algorithm. In contrast, feature selection algorithms are generic because they only provide an answer to the question of how many of the best features are relevant and should be retained in the subsequent data analysis or interpretation of the results, which is often arbitrary, such as taking the top 5 or 10. The choice of feature selection method is independent of the cABC analysis and is at the discretion of the data analyst. If a flawed feature selection method has assigned inflated importance values to irrelevant variables, cABC analysis cannot correct this. An example is Gini impurity-based feature importance, available as a default method in some random forest software packages, which measures how effective the feature is at reducing uncertainty when constructing decision trees based on the mean reduction in impurity (or "Gini importance").Its use is now discouraged, as it has been shown to occasionally produce biased results with inflated importance of numerical features that are not predictive for unseen data^57,58^. In the documentation of the "scikit-learn" package (https://scikit-learn.org/stable/^40^), an example is given where two random variables, one categorical and one numerical, were added to a dataset on survival factors in the sinking of the Titanic (https://scikit-learn.org/stable/auto_examples/inspection/plot_permutation_importance.html). We have used this published code and added the present cABC analysis. Using the Gini feature importance on independent data separated before random forest training, d = 4 features were selected for the ABC subset "A", while based on the importance measures obtained using permutation importance as the recommended alternative feature selection method, only gender was selected. However, this may be a coincidence, and in other scenarios the importance values for relevant and nonsensical variables may be closer. Other diagnostic tools must then be used, such as evaluating the classification performance when the unselected features are used for training instead of the selected features, as recently suggested^59^. In the present example, the permutation importance-based feature proved sufficient to train a random forest classifier to assign unseen cases (20% separated before feature selection and training) to classes with balanced accuracy similar to that using the full feature set. The classifier trained with the unselected features was significantly less powerful. In contrast, random forests trained with the Gini impurity-based faulty feature set performed worse, and importantly, the classification accuracy of random forests trained with the unselected features of this variant was closer to that of those trained with the selected features (Fig. 6).

## Conclusions

ABC methods, such as those presented here, are essential in data analysis today to identify simple, easy to understand (the critical few) properties of complex multivariate data. The selection of the k best items is often decided heuristically or arbitrarily. The cABC analysis provides this number calculated from actual data. It uses the point on the cumulative importance of items that is closest to the "one gets it all" extreme, i.e., a single item already contains all the relevant information. In addition, cABC analysis, in its recursive variant, provides a computational means of reducing information to a bare minimum, thereby increasing human comprehension.

## Data Availability

The cABC method is implemented in the Python package "cABCanalysis" available at https://pypi.org/project/cABCanalysis/.
